# Pretransplant BKV-IgG serostatus and BKV-specific ELISPOT assays to predict BKV infection after kidney transplantation

**DOI:** 10.3389/fimmu.2023.1243912

**Published:** 2023-09-21

**Authors:** Hyunjoo Bae, Seungwon Jung, Byung Ha Chung, Chul Woo Yang, Eun-Jee Oh

**Affiliations:** ^1^ Department of Biomedicine & Health Sciences, Graduate School, The Catholic University of Korea, Seoul, Republic of Korea; ^2^ Department of Laboratory Medicine, Uijeongbu Paik Hospital, Uijeongbu, Republic of Korea; ^3^ Division of Nephrology, Department of Internal Medicine, Seoul St. Mary’s Hospital, College of Medicine, The Catholic University of Korea, Seoul, Republic of Korea; ^4^ Resesarch and Development Institute for In Vitro Diagnostic Medical Devices, College of Medicine, The Catholic University of Korea, Seoul, Republic of Korea; ^5^ Department of Laboratory Medicine, Seoul St. Mary’s Hospital, College of Medicine, The Catholic University of Korea, Seoul, Republic of Korea

**Keywords:** BK polyomavirus, kidney transplant recipients, pre-transplant BKV-IgG serostatus, BKV-specific ELISPOT, BK viremia

## Abstract

**Introduction:**

Polyomavirus (BKV) infection can lead to major complications and damage to the graft in kidney transplant recipients (KTRs). We investigated whether pretransplant BK serostatus and BK-specific cell-mediated immunity (CMI) predicts post-transplant BK infection.

**Methods:**

A total of 93 donor-recipient pairs who underwent kidney transplantation (KT) and 44 healthy controls were examined. Assessment of donor and recipient BKV serostatus and BKV-CMI in recipients was performed prior to transplantation using BKV-IgG ELISA and BKV-specific IFN-g ELISPOT assays against five BK viral antigens (LT, St, VP1, VP2, and VP3). BK viremia was diagnosed when blood BKV-DNA of 104 copies/mL or more was detected during follow-up periods.

**Results:**

Anti-BKV IgG antibody was detected in 74 (79.6%) of 93 KTRs and in 68 (73.1%) of 93 KT donors. A greater percentage of KTRs who received allograft from donors with high levels of anti-BKV IgG had posttransplant BK viremia (+) than KTRs from donors with low anti-BKV IgG (25.5% [12/47] vs. 4.3% [2/46], respectively; P = 0.007). Pretransplant total BKV-ELISPOT results were lower in BK viremia (+) patients than in patients without viremia (-) 20.5 [range 9.9−63.6] vs. 72.0 [43.2 - 110.8]; P = 0. 027). The sensitivity and specificity of the total BKV-ELISPOT assay (cut-off ≤ 53 spots/3×105 cells) for prediction of posttransplant BK viremia were 71.4 (95% CI: 41.9–91.6) and 54.4 (42.8–65.7), respectively. The combination of high donor BKV-IgG, low recipient BKV-IgG, and low total BKV-ELISPOT results improved specificity to 91.1%.

**Discussion:**

Our study highlights the importance of pretransplant BKV-IgG serostatus and BKV-specific CMI in predicting posttransplant BKV infection in KTRs. The combination of high donor BKV-IgG, low recipient BKV-IgG, and low total BKV-ELISPOT results predicted BK viremia after KT. Pretransplant identification of patients at highrisk for BK viremia could enable timely interventions and improve clinical outcomes of KTRs.

## 1 Introduction

The BK polyomavirus (BKV) is an opportunistic pathogen of the Polyomaviridae family. After an initial respiratory tract infection with BKV in childhood, which is frequently mild or asymptomatic, the virus persists in a latent state primarily within the renal tubules and epithelial cells of the urinary tract (1–3). Immunosuppressive therapy increases the risk of reactivation in kidney transplant recipients (KTRs) (4, 5). Reactivation of latent BKV in KTRs can lead to BKV-associated nephropathy (BKVAN) and is associated with an increased risk of allograft dysfunction or graft failure in up to 30% of cases (6). Currently, no effective antiviral drugs for BKV infection are available, so the primary strategy for managing the BKV infection is to reduce immunosuppression and restore the immune response against BKV; however, reducing immunosuppressive therapy to limit BKVAN can increase the risk of allograft rejection.

Several posttransplant BKV monitoring strategies have been implemented to improve the management of BKV replication in the immunocompromised host. The most widely used early diagnostic screening method is to periodically measure BKV DNA in the blood and urine up to 1 year after transplantation using quantitative PCR. Despite improvements in screening methods based on regular monitoring of the viral load, the incidence of BKVAN has remained stable over the last few years (5). Therefore, additional indicators to predict the progression of BKV infection are needed for to improve treatment outcomes of KTRs.

Monitoring of BKV-specific humoral and cellular immunity has been considered to assess the risk of BKV infection. However, previous studies have shown that the predictive value of pretransplant BKV-IgG antibodies in KTR is uncertain (2, 6–11). Recently, antibodies against the BKV capsid protein VP1 have been reported to have subtype-specific virus-neutralizing activity in KTRs (12). Some studies have reported that recipient and donor BKV serostatus could be an important predictor of BKV reactivation (2, 13). Since BKV in KTRs may be of donor origin, it is important to assess the donor’s serostatus and BKV titer as risk factors for posttransplant BKV infection (13–15). Recently, Saláková et al. reported that the donor BKV-IgG seroprevalence and antibody level were strongly associated with posttransplant BK viremia and BKVAN in a study of 210 KTRs and 130 donors (16). Finally, they confirmed recommendation by Hirsh and Randhawa that KTRs from seropositive donors should be routinely screened for BKV-DNA in plasma much more frequently than KTRs from seronegative donors (5). Regarding the recipient BKV-IgG, Ginevri et al. suggested that the negative antibody status of the recipient was the most substantial predictor of BKV infection in pediatric KTRs (17). Another study has also highlighted the significant association between the recipient’s seronegative status for BK viremia and the development of BKVAN (18).

Several studies have assessed BKV-specific cellular immunity (CMI) in KT patients, although typically only after transplantation. An increase in BKV-specific T cells has been associated with resolution of BK viremia, and cellular immunity has been reported to play an important protective role in regulating BKV proliferation and progression of BKVAN (6, 8, 11, 19–21). In addition, low pretransplant BKV-specific T cells and posttransplant BKV-CMV unresponsiveness or loss have been reported to be associated with BK viremia and BKVAN (6, 22). Notably, a recent study has underscored the diminished proliferative response of BKV-specific CD8 T cells as a contributing risk factor for BKVAN (23).

Given the importance of pre-transplant immunity in preventing post-transplant BKV infection, screening both humoral and cellular immunity before transplantation may be an effective measure to predict and prevent BKV infection. In the present study, we assessed the BKV-IgG serostatus of kidney donors and recipients, and also measured BKV-specific ELISPOT responses in KTRs prior to transplantation, which had not been previously investigated. The aim of this study was to identify pretransplant risk factors for posttransplant BKV infection by assessing BKV-specific humoral and cellular immunity.

## 2 Material and methods

### 2.1 Study population

This retrospective study included 93 KTRs who consented to donate peripheral blood at the time of their pretransplant cross-matching test. All KTRs underwent kidney transplantation at Seoul St. Mary’s Hospital between January 2016 and October 2019. Pre-transplant serum was also collected from 93 matched donors, and all sera were collected no more than 1 month prior to the kidney transplant. The inclusion criteria were adults aged 20 years or older with available pretransplant blood sampling from both donors and recipients. Patients who had received organ transplants other than the kidney or who did not provide informed consent for the study were excluded. An additional 44 healthy donors were included as controls. This study was approved by the Institutional Review Board at Seoul St. Mary’s Hospital (KC19TESI0043).

### 2.2 Diagnosis of BK viremia and BKVAN

The diagnosis of BKV infection and management of immunosuppression in KTRs were performed according to the protocol of our transplant center (11). Donor and recipient BKV viral loads in whole blood were measured using a Real-Q BK Quantification Kit (Biosewoom, Seoul, Korea) to detect the viral capsid protein (VP-1) gene using an ABI PRISM 7000 real-time PCR system (Applied Biosystems). The recipient’s BKV viral load was monitored at 1, 3, 6, 9, and 12 months after transplantation, and then once a year thereafter or when kidney function was impaired. BK viremia was diagnosed when BKV-DNA of 10^4^ copies/mL or more was detected during the follow-up period (median 19 [range 18−29) months), and therapeutic intervention was initiated to prevent progression to BKVAN. In the case of BK viremia patients, monitoring using quantitative PCR was performed until BK viremia resolved, and mycophenolate mofetil (MMF) treatment was discontinued. If BK viremia persisted after 1 month, a transplant biopsy was performed, and the dose of calcineurin inhibitor was reduced by 20% while leflunomide was started. A further 2 weeks of BK viremia resulted in a further 50% reduction in tacrolimus. If BK viremia persisted beyond this period, tacrolimus was switched to sirolimus. If BK viremia still persists or increases after an additional 2 weeks, a 5-day course of intravenous immunoglobulin (IVIG) for BKVAN was administered.

In patients with BK viremia, monitoring using quantitative PCR was carried out until BK viremia resolved, and mycophenolate mofetil (MMF) treatment was stopped. If BK viremia persisted after 1 month, a graft biopsy was performed, calcineurin inhibitors were reduced by 20%, and leflunomide was initiated. The diagnosis of BKVAN was made when the following criteria were met: (1) a basophilic intranuclear viral inclusion body suggestive of BKVAN was observed; (2) immunochemical staining for SV40T antigen was positive; (3) there was evidence of BKV proliferation in at least one of the urine cell test, urine PCR, and/or blood PCR results.

### 2.3 BKV-specific IgG ELISA

Anti-BKV IgG antibody was assessed using the ELISA-VIDITEST anti-BKV IgG kit (VIDIA, Ltd., Czech Republic), which employs recombinant species-specific BKV antigens and targets major BKV type I and IV genotypes for detection. Serum samples were stored at −80°C until use. Diluted serum was dispensed into wells coated with recombinant BKV-specific antigens and incubated for 30 minutes at room temperature. After washing, a secondary antibody (anti-human IgG antibody) labeled with peroxidase was added and incubated at room temperature for 30 minutes. The reaction was detected with 100 μL of 3,3’,5,5’-tetramethyl benzidine substrate solution at room temperature for 10 minutes. The optical density (OD) of each well was measured at 450 nm. The cut-off value was determined by multiplying the mean absorbance of the indicated calibrator by the correction factor specified in the quality control certificate.

### 2.4 BKV-specific IFN- γ ELISPOT

A BKV-specific human IFN-γ ELISPOT (BKV-ELISPOT; ELISPOT Ready-SET-Go kit (eBioscience, San Diego, CA, USA) assay specific for each of the five BKV structural proteins (LT, St, VP1, VP2 and VP3) was performed as described in a previous study (11). Briefly, 96-well ELISPOT plates (Millipore, Cat. No. MAIPS4510) were coated with diluted anti-IFN-γ monoclonal capture antibody at 4°C overnight. After washing the microplate twice with the coating buffer, 200 uL of RPMI-1640 culture solution containing fetal bovine serum and 1% penicillin as a blocking medium was dispensed and left at room temperature for 1 hour. Peripheral blood mononuclear cells (PBMCs) were freshly isolated from heparin-treated whole blood samples by density gradient centrifugation using a Ficoll solution. The PBMCs (3 x 10^5^cells/well) were stimulated with phorbol 12-myristate 13-acetate (PMA)/ionomycin as a positive control, RPMI medium as a negative control, and PepMix BKV (1 µg/mL of LT, st, VP1, VP2, and VP3; JPT Peptides Technologies, Berlin, Germany) at 36°C in a CO_2_ incubator for 24 hours. After washing the microplate, 100 µL/well of the detection antibody (biotin-labeled anti-human IFN-γ antibody) was dispensed and reacted at room temperature for 2 hours. Further processing was performed according to the manufacturer’s instructions. Spot on the dried microplate were counted using an ELISPOT image analyzer (Cellular Technologies Ltd., Cleveland, OH, USA). PBMCs were evaluated in duplicate in each ELISPOT assay and average numbers of spots detected per 3×10^5^ PBMCs. Spot numbers were calculated by subtracting the in the negative control from the average number of spots in the test wells. BKV-ELISPOT (sum of the five peptide-specific ELISPOT) results were also calculated.

### 2.5 Statistical analysis

Counts and percentages were calculated for categorical data, and the data were analyzed using the chi-square test and Fisher’s exact test. The median and 95% confidence interval (95% CI)), were calculated for continuous data, and the data were compared using Student’s t-test and the Mann-Whitney U test. Receiver operating curve (ROC) analyses were used to define the cut-off number of spots for the BKV-ELISPOT assay to predict BKV infection. All statistical analyses were performed using MedCalc^®^ Statistical Software version 20.218 (MedCalc Software Ltd, Ostend, Belgium; https://www.medcalc.org) and GraphPad prism version 9.0 for Windows (GraphPad Software, San Diego, California USA). All statistical analyses were two-tailed tests, and a P value less than 0.05 was defined as statistically significant.

## 3 Results

### 3.1 Baseline characteristics of kidney transplant recipients with respect to BK viremia

Among the 93 KTRs, 14 (15.1%) developed BK viremia and 5 (5.4%) were diagnosed with BKVAN. The mean recipient age was 48.4 years (ranging from 20 to 67 years) and 63.4% of recipients were male. The baseline characteristics of the study participants with respect to BK viremia are summarized in **Table 1**. In the BK viremia (+) group, both donors and recipients were older than those without BK viremia (-) group; P = 0.028 and P = 0.036, respectively). The incidence of new-onset BK viremia after transplantation was significantly greater in recipients who had primary kidney disease due to hypertension (P = 0.009) or polycystic kidney disease (P = 0.042). There were no significant differences in the baseline characteristics and percentages of recipients who rejected the transplanted kidney between BK viremia (+) and BK viremia (-) patients.

**Table 1:** Demographic characteristics and BK polyomavirus viremia status of the study kidney transplant (KT) recipients.

Characteristics	Total		BK viremia (+)		BK viremia (-)		P value
KT Recipients, n (%)	93		14 (15)		79 (85)		
Primary renal disease, n (%)							
DM	22 (23.7)		2 (14.3)		20 (25.3)		NS
Hypertension	7 (7.5)		4 (28.6)		3 (3.8)		0.009
Polycystic kidney disease	6 (6.5)		3 (21.4)		3 (3.8)		0.042
GN	33 (35.5)		3 (21.4)		30 (38.0)		NS
Unknown	25 (26.9)		2 (14.3)		23 (29.1)		NS
Age at transplantation (years ± SD)	48.4 ± 10.7		54.1 ± 11.3		47.4 ± 10.3		0.028
Gender, male, n (%)	59 (63.4)		12 (85.7)		47 (59.5)		NS
Blood incompatible, n (%)	42 (45.2)		9 (64.3)		33 (41.8)		NS
HLA mismatch number, mean ± SD	4 ± 1.7		4 ± 1.7		3.5 ± 1.7		NS
Prior kidney transplant, n (%)	9 (9.7)		1 (7.1)		8 (10.1)		NS
PRA at pre-KT, n (%)	29 (31.2)		4 (28.6)		25 (31.6)		NS
DSA at pre-KT, n (%)	14 (15.0)		5 (35.7)		9 (11.4)		NS
Induction therapy, n (%)							
Basiliximab	72 (78.5)		11 (78.6)		62 (78.5)		NS
Anti-thymocyte globulin	21 (22.6)		3 (21.4)		18 (22.8)		NS
KT Donor							
Age, years + SD	46.0 ± 11.8		52.1 ± 7.2		44.9 ± 12.2		0.036
Gender, male, n (%)	42 (45.2)		7 (50.0)		35 (44.3)		NS
Deceased donor, n (%)	3 (3.2)		1 (7.1)		2 (2.5)		NS
Acute rejection, n (%)							
AAMR	4 (4.3)		1 (7.1)		3 (3.8)		NS
ATCMR	10 (10.8)		3 (21.4)		7 (8.9)		NS
CAMR	1 (1.1)		0 (0.0)		1 (1.3)		NS
Pretransplant anti-BKV seropositivity							
in donors, n (%)	68 (73.1)		13 (92.9)		55 (69.6)		NS
in recipients, n (%	74 (79.6)		9 (64.3)		65 (82.3)		NS
Pretransplant anti-BKV IgG levels (OD)							
in donors, median (95% CI)			1.80 (1.63-2.65)		1.14 (0.67-1.63)		0.014
in recipients, median (95% CI)			2.14 (0.26-3.11)		1.66 (1.21-2-17)		NS

KT, kidney transplantation; DM, diabetes mellitus; GN, glomerulo-nephritis; BKV, BK polyomavirus; DSA, donor-specific antigen; PRA, panel-reactive antibody; HLA, human leukocyte antigen; AAMR, acute antibody-mediated rejection; ATCMR, acute T cell-mediated rejection; CAMR, chronic active antibody-mediated rejection; NS, not significant (P > 0.05).

### 3.2 Pretransplant anti-BKV-specific IgG seropositivity in donors and recipients

Using the cut-off level provided by the manufacturer, the anti-BKV IgG antibody assay was positive in 74 (79.6%) of 93 KTRs and in 68 (73.1%) of 93 KT donors and 31 (70.5%) of 44 healthy controls. There was no significant difference in seropositivity between the 93 KTR and 137 healthy participants (P > 0.05). Next, we analyzed the incidence of post-KT BK viremia according to pre-KT donor or recipient (D or R) anti-BKV serostatus. While not statistically significant, a greater percentage of KTRs who received transplants from seropositive donors (D+) were subsequently diagnosed with BKV infection than those who received transplants from seronegative donors (D−; 19.1% [13/68] vs. 4.0% [1/25]; P = 0.102). The anti-BKV serostatus of KTRs was not associated with post-transplant BK viremia (R+, 12.2% (9/74) vs. R-, 26.3% (5/19); P > 0.05). Five recipients developed BKVAN: three were D+/R−, one was D+/R+, and one was D−/R+.

### 3.3 Pretransplant anti-BKV-specific IgG levels in donors and recipients

**Figure 1:**
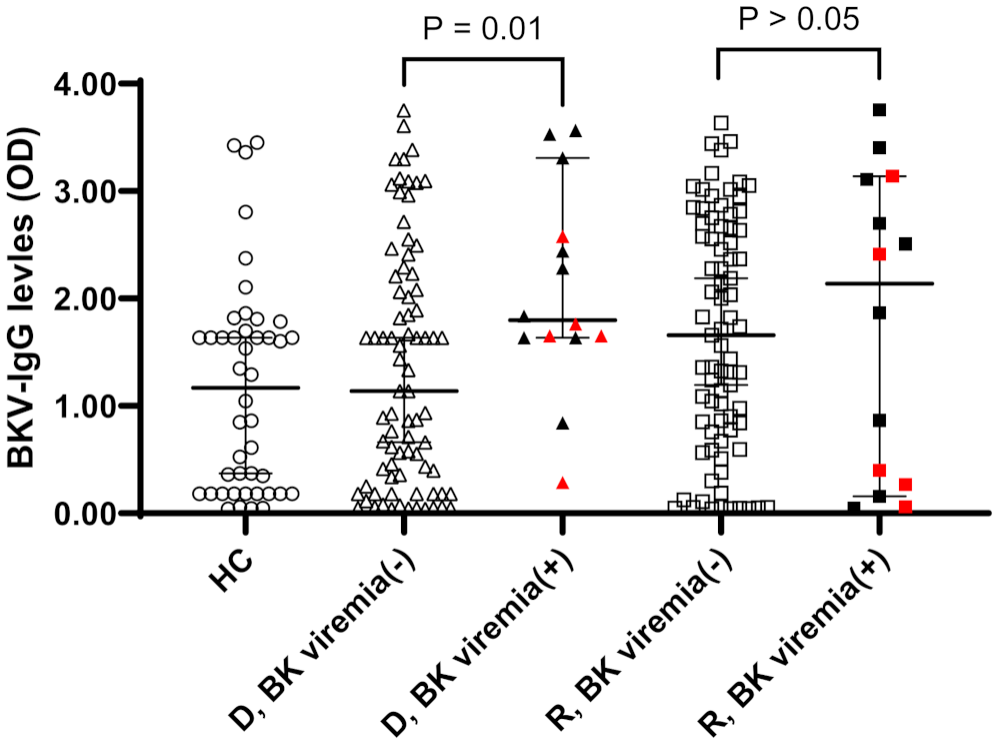
Anti-BKV IgG serostatus (optical density, OD) in 44 healthy controls (HC) and 93 donor (D) and recipients (R) prior kidney transplantation. Donors and recipients were stratified according to the development of posttransplant BK viremia development (BK viremia+) or not (BK viremia-). Donors from BK viremia (+) had higher OD values than donors from BK viremia (-) group (P = 0.01). Red symbols indicate results from five patients with BKV-associated nephropathy.

**Figure 2:**
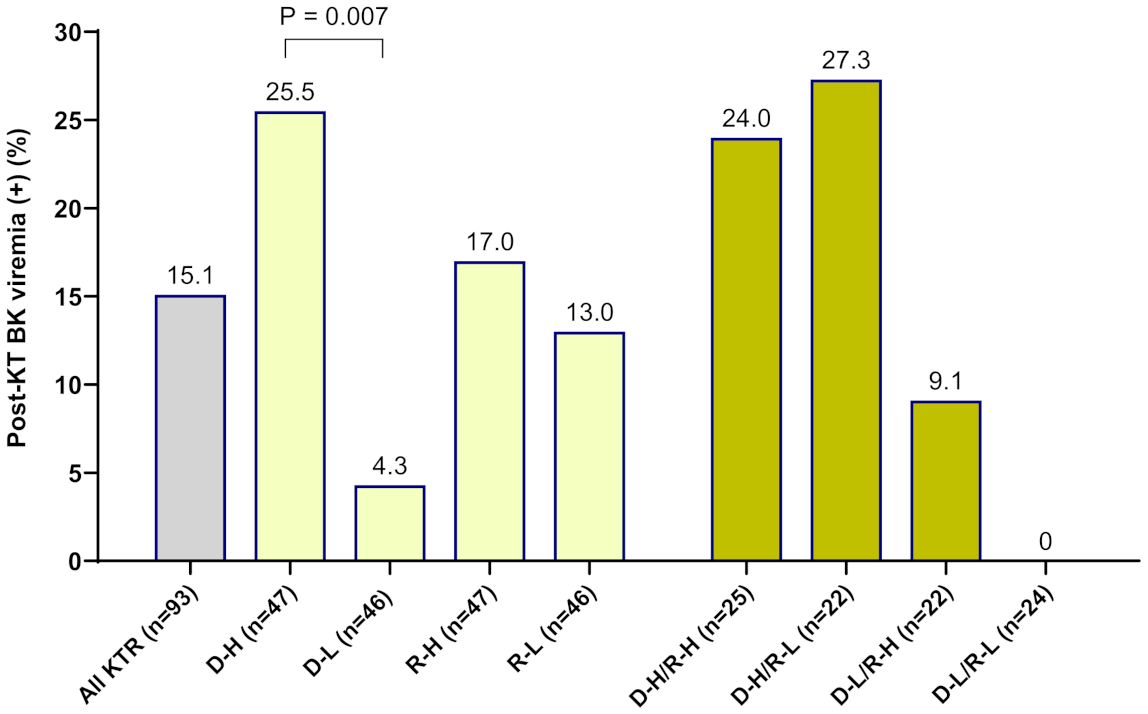
The incidence of new-onset posttransplant BK viremia was analyzed in 93 kidney transplant recipients (KTRs) according to the pretransplant anti-BKV-IgG serostatus in donors and recipients. Among KTRs, a greater percentage of the high-anti-BKV-IgG donor (D-H) group had posttransplant BK viremia (+) than those in the low-anti-BKV-IgG donor (D-L) group (P = 0.007). Outcomes were also assessed with respect to recipient anti-BKV-IgG serostatus (high anti-BKV-IgG, R-H; low anti-BKV-IgG, R-L). When examining donor/recipient pairs, the incidence of post-transplant BK viremia (+) was 24.0% (6/25) in the D-H/R-H group and 27.3% (6/22) in the D-H/R-L group.

When OD values of BKV-IgG results instead of positivity were analyzed, donors in the BK viremia (+) group had higher anti-BKV-IgG OD values than those in the BK viremia (-) group (median 1.80 [95% CI 1.63-2.65] vs. 1.14 [95% CI 0.67-1.63], P = 0.014; **Figure 1**). The recipients’ anti-BKV-IgG levels were not associated with development of post-KT BK viremia (P > 0.05). Considering the high incidence of anti-BKV-IgG seropositivity, we categorized the KTRs with respect to anti-BKV IgG as those with high OD values (R-H; n = 47, OD range 1.63–2.28) and low OD values (R-L; n = 46, OD range; 0.04−1.56). Donors were also divided into two groups according to anti-BKV IgG levels as D-H (n = 47, OD range 1.63−3.75) and D-L (n = 46, OD range 0.04−1.56). A greater percentage of KTRs who received a kidney from the D-H group had posttransplant BK viremia than those who received one from the D-L group (25.5% [12/47)] vs. 4.3% [2/46], P = 0.007; **Figure 2**). Among fourteen BK viremia (+) recipients, 12 (85.8%) received transplants from the high anti-BKV IgG (D-H) group. Among the five patients with BKVAN, four received transplants from D-H donors. When examining D/R pairs, the incidence of BK viremia (+) increased from 4.3% (2/46) in the D-L group to 24.0% (6/25) in the D-H/R-H group and 27.3% (6/22) in the D-H/R-L group.

### 3.4 Pretransplant BKV-specific INF-γ ELISPOT results

We employed a BKV-specific ELISPOT assay of five BKV peptides in the 93 pretransplant KTRs and 44 healthy controls. The results showed a trend toward higher and wider ranges of BKV-ELISPOT values in the KTRs compared to healthy controls (**Supplementary Figure 1**). Of the five peptide-specific ELISPOT results, the VP2-ELISPOT results (spots/3×10^5^ PBMCs) were higher in KTRs compared to healthy controls (median 8.5 [95% CI 6.0−13.0] vs. 5.0 [4.0−8.0], P = 0.040).

**Figure 3:**
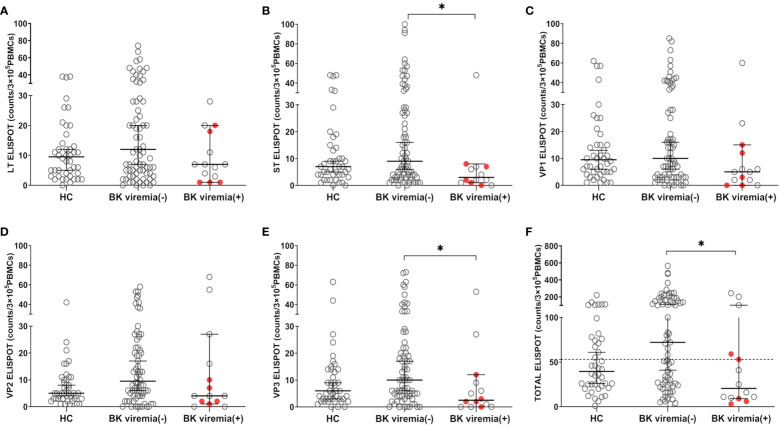
Comparisons of pretransplant BKV-ELISPOT assay results for BKV-specific peptides (LT, ST, VP1, VP2, VP3, and peptide totals) in the healthy control (HC), posttransplant-BK viremia (+), and no-posttransplant BK viremia (-) groups after kidney transplantation (KT). The BKV-associated nephropathy group is represented by a red-filled circle symbol. The long line represents the median and short line represents the 95% CI. The dashed line represents the cut-off value of 53 spot numbers/3 x 10^5^ cells. BK viremia (+) KT recipients had significantly lower ST**(B)**, VP3**(E)**, and TOTAL**(F)** ELISPOT results than those in the BK viremia (-) group. *P<0.05. CI, confidence interval.

When comparing the pre-transplant BKV-ELISPOT results between posttransplant BK viremia (+) and BK viremia (-) patients, BK viremia (+) patients showed a trend toward lower ELISPOT results for all five BKV peptides compared to BK viremia (-) patients, with significant differences for the St- and VP3-ELISPOT results (St-ELISPOT 3.0 [1.0 - 7.1] vs. 9.0 [6.0 -16.0], P = 0.038; VP3-ELISPOT 2.5 [0.0 - 9.3] vs. 10.0 [6.0 - 17.0], P = 0.013; **Figure 3**). The total BKV-ELISPOT results (sum of the five peptide-specific ELISPOT results) were also lower in BK viremia (+) patients compared to BK viremia (-) patients (20.5 [9.9 - 63.6] vs. 72.0 [43.2 - 110.8], P = 0. 027; **Figure 3F**). The five patients who developed BKVAN had low total BKV-ELISPOT results of 3, 6, 9, 53 and 59 spots/3×10^5^ PBMCs.

Analysis of receiver operating curve (ROC) plots for pretransplant total BKV-ELISPOT results to predict posttransplant BK viremia showed an area under the curve (AUC) of 0.686 (95% CI: 0.581 – 0.778, P = 0.022). At the optimal cut-off value of ≤ 53 spots/3×10^5^ cells, the sensitivity and specificity of total BKV-ELISPOT for predicting posttransplant BK viremia were 71.4 (95% CI: 41.9 – 91.6) and 54.4 (42.8 – 65.7). The overall positive predictive (PPV) and negative predictive (NPV) values of the total BKV-ELISPOT results were 21.7% (95% CI: 15.6 – 29.5) and 91.5 (95% CI: 82.1 – 96.2), respectively.

### 3.5 Prediction of posttransplant BK viremia (+) by combination of pretransplant anti-BKV-IgG levels and BKV-ELISPOT results

**Figure 4:**
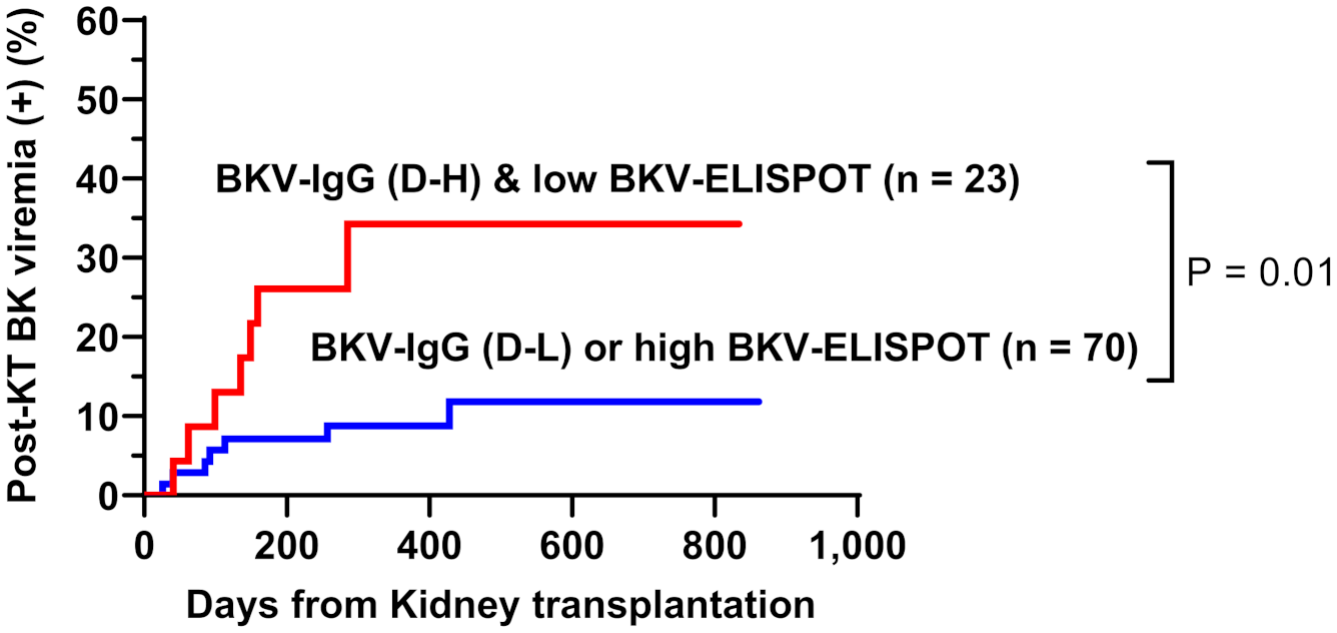
Cumulative incidence of posttransplant BK viremia in kidney transplant recipients stratified by donor pretransplant anti-BKV-IgG serostatus (H, high anti-BKV-IgG levels; L, low anti-BKV-IgG levels) and recipient total BKV-ELISPOT result. Recipients who had low BKV-ELISPOT results and received a kidney from high anti-BKV-IgG donors developed posttransplant BK viremia more frequently than the other patient groups.

**Figure 5:**
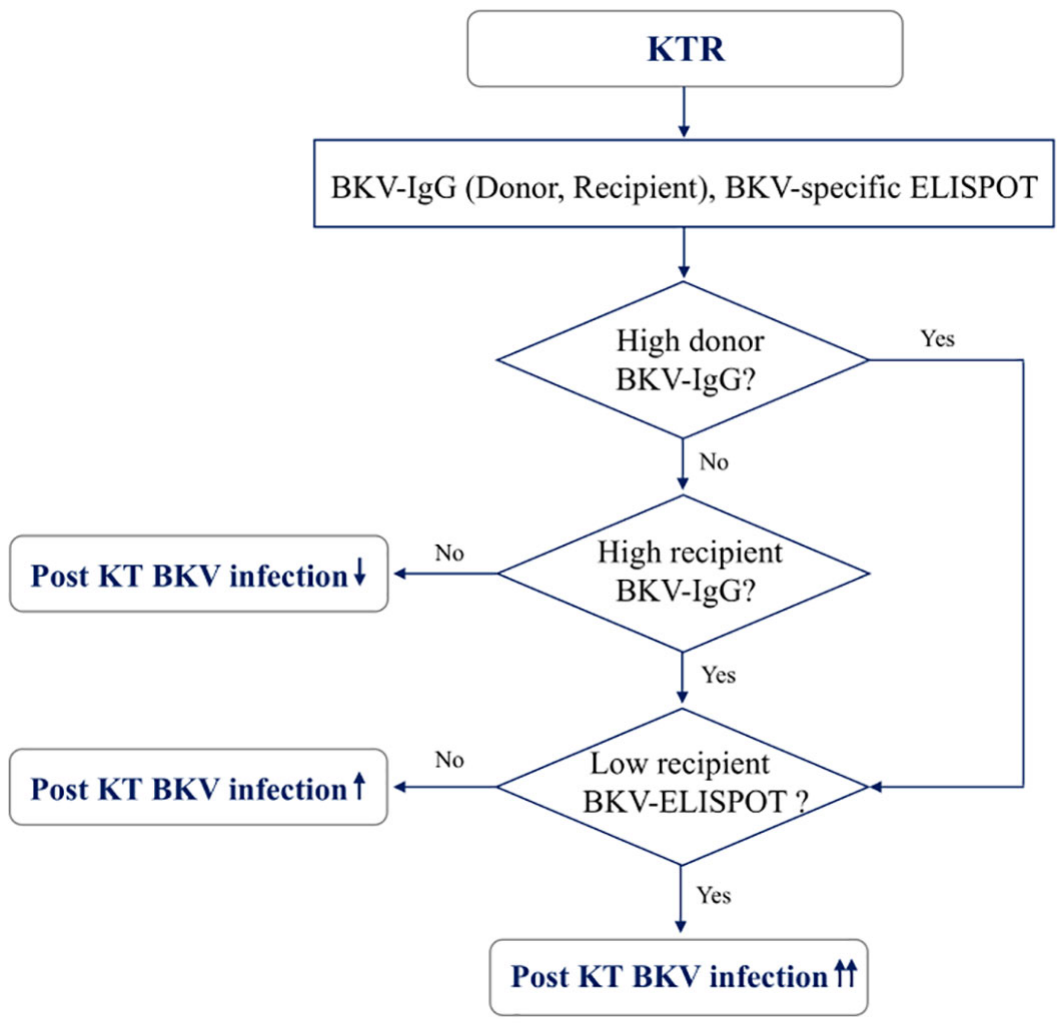
A model for predicting risk of BKV infection after transplantation in kidney transplant recipients based on pre-transplant BKV-IgG serostatus and BKV-specific ELISPOT results.

When total BKV-ELISPOT results before transplant were compared with anti-BKV-IgG levels in the 93 KTRs, there was no correlation between the two values. The elevated pre-transplant BKV-ELISPOT results in some seronegative patients provided additional evidence that the two risk factors were independent (**Supplementary Figure 2**). Diagnostic values were determined by combining pretransplant donor/recipient anti-BKV-IgG levels and recipient total BKV-ELISPOT results. For the posttransplant BK viremia prediction, the combination of high donor anti-BKV-IgG (D-H), low recipient BKV-IgG (R-L) and low recipient total BKV-ELISPOT results improved specificity to 91.1%. In case of decreased total BKV-ELISPOT results (≤ 53 spots/3×10^5^ PBMCs) or high donor anti-BKV IgG levels, the NPVs for protection from posttransplant BK viremia was 100% (**Table 2**). Kaplan-Meier analyses demonstrated greater incidence of BK viremia in patients who had lower pretransplant total BKV-ELISPOT results and received a transplant from a donor with high anti-BKV-IgG levels compared with patients who did not (P = 0.003; **Figure 4**). We suggested an algorithm to predicts the risk of posttransplant BKV infection in KTRs based on pre-transplant BKV-IgG serostatus BKV-specific ELISPOT results (**Figure 5**).

**Table 2:** Diagnostic accuracy of pretransplant donor and recipient anti-BKV-IgG levels and recipient BKV-ELISPOT results in kidney transplant recipients for prediction of BK viremia (>10^4^ copies/mL) after kidney transplantation in the study population.

	Sensitivity % (95% CI)	Specificity % (95% CI)	PPV % (95% CI)	NPV % (95% CI)
High donor anti-BKV-IgG	85.7 (57.2-98.2)	55.7 (44.1-66.9)	25.5 (14.4-40.6)	95.7 (84.0-99.2)
Low recipient BKV-ELISPOT*	71.4 (41.9-91.6)	54.4 (42.8-65.7)	21.7 (15.6-29.5)	91.5 (82.1-96.2)
High donor anti-BKV-IgG and low recipient BKV-ELISPOT	57.4 (28.9-82.3)	79.8 (69.2-88.0)	33.3 (21.0-48.4)	91.3 (85.0-95.1)
High donor anti-BKV-IgG, and low recipient anti-BKV-IgG and BKV-ELISPOT	35.7 (12.8-64.9)	91.1 (82.6-96.4)	41.7 (20.9-65.9)	88.9 (84.3-92.2)
High donor anti-BKV-IgG or low recipient BKV-ELISPOT	100.0 (76.8-100.0)	30.4 (20.5-41.8)	20.3 (18.0-22.7)	100.0 (-)

*total BKV-ELISPOT result with spot number ≤ 53/3 x 10^5^ PBMCs.

PPV, positive predictive value; NPV, negative predictive value; CI, confidence interval.

## 4 Discussion

The BK polyomavirus is an opportunistic organism that frequently infects immunosuppressed patients after kidney transplantation, with BKVAN being a notable cause of impaired kidney function that may progress to transplant failure in up to 50% of cases (24, 25). Pretransplant risk factors associated with reactivation of BKV have been reported, including anti-thymoglobulin, administration of enhanced immunosuppressants, history of previous acute rejection, old age, HLA mismatch, HLA-C7 deficiency and anti-BKV IgG positivity (26). In the present study, pretransplant risk factors were evaluated using donor/recipient anti-BKV IgG levels and recipient BKV ELISPOT results to predict post-transplant BKV infection.

The BK virus, which causes infection in KTRs, could theoretically originate from both kidney donors and recipients (27). Anti-BKV IgG seropositivity through the manufacturer’s suggested cut-off levels was 79.6% in KTR and 72.3% in healthy participants, which are similar to the previous study (28). Regarding the association between pretransplant anti-BKV IgG results and posttransplant BKV infection, in a previous study, KTRs who were negative for anti-BKV IgG had four-fold greater incidence of posttransplant BKV infection when the donor was positive for anti-BKV IgG, compared with donors that were negative for anti-BKV IgG (29). Several studies have reported a significantly higher risk of BKV infection in patients transplanted from anti-BKV-IgG-positive donors (14, 30, 31). However, Hirsch et al. suggested that the D+/R− group is not the highest-risk group (28), and the significance of the donor or the recipient anti-BKV antibody serostatus is still uncertain. In the present study, there was a trend toward a greater percentage of KTRs who received allograft from seropositive donors (D+) developing BK viremia, but the pretransplant serostatus of donor and recipients was not significantly associated with posttransplant BK viremia (p >0.05). However, when anti-BKV IgG levels were examined instead of seropositivity, the D-H/R-L group had the greatest risk of developing BK viremia (p <0.001). These findings are consistent with the those of Saláková et al. (16) who established a significant correlation between donor seropositivity and recipient post-transplant BKV infection. A trend toward donor seropositivity and BKVAN was observed in their study, but lacked statistical significance. However, when assessing BKV IgG levels, both post-transplant BKV infection and BKVAN were more prevalent in recipients with higher donor BKV-IgG levels. They also highlighted a significantly increased risk of BKV infection after transplantation in the D+/R- group. In terms of BKVAN, four out of five BKVAN patients received transplants from the D-H group. These results are consistent with previous studies suggesting that the high risk of posttransplant BKV infection is not limited to the D+/R- group and that anti-BKV IgG levels in both donors and recipients may play an important role (28, 30, 32). Our data consistent with those of previous studies indicating that transplantation from donors with high anti-BKV IgG levels (D-H) into recipients with low anti-BKV IgG levels (R-L) is an independent risk factor for BKV infection after transplantation by Dakroub et al. (10, 13). Smith et al. (18) also reported that BKV seronegative in 173 pediatric kidney transplant recipients was strongly associated with the incidence of BKVAN after transplantation. These findings suggest the importance of standardized anti-BKV-IgG testing and of clear guidelines for evaluation of anti-BKV-specific IgG levels and the definition of seropositivity. Currently, pretransplant screening of anti-BKV serostatus in kidney donors and recipients is neither mandatory nor routinely performed. Further studies are required to investigate the relationships between posttransplant BK viremia and pretransplant anti-BKV IgG antibody levels measured using standardized assays.

In terms of cellular immunity, BKV-specific T cells play an important role in the control of BKV infection by maintaining a persistent memory T cell response to the virus (19, 33–35). A prior meta-analysis reported that ELISPOT assays could be an effective tool to assess BKV-specific cellular immunity (7). In previous studies, posttransplant monitoring of BKV-specific cellular immune responses were conducted to investigate their associations with posttransplant reactivation of BKV (34, 36, 37). However, there have been few studies assessing whether pretransplant BKV-specific immunity levels can predict posttransplant BKV reactivation. Mutlu et al. (8) analyzed BKV-specific CD4+ T-cell responses of KTRs before transplantation and did not show any association between pre-transplant response and BKV reactivation, like the other two previous studies (6, 38). However, they confirmed that clearance of BK viremia or decrease of viral load corresponds with an increase in BKV-specificCD4+T-cellresponse. Other reports have also suggested that the absence of or low response of BKV-specific T cells may be a risk factor for BKV reactivation, while others have found no clear association (6, 8, 39). Dekeyser et al. found a gradual loss of BKV-specific T-cell polyfunctionality with BKV reactivation, particularly in patients with BKVAN. This exhaustion of BKV-specific T-cell responses is linked to impaired control of viral replication in the kidney (23). In present study, KTRs tended to have greater and broader BKV-ELISPOT results before transplantation compared to healthy controls, with a statistically significant difference for VP2-ELISPOT. Next, we investigated whether the pretransplant BKV-ELISPOT results could predict the development of posttransplant BK viremia. In our study, the 14 BK viremia (+) patients tended to have lower pretransplant ELISPOT responses to all 5 BKV peptides than BK viremia (-) patients. Among the five peptide-specific ELISPOTs, significant differences were observed for the St, VP3 and total BKV-ELISPOT results. The ROC analysis of total BKV-ELISPOT results showed an AUC of 0.686 and sensitivity of 71.4% and specificity of 54.4% using the cut-off value of ≤53 spots/300,000 cells. Also, four of five BKVAN patients had decreased pretransplant BKV-ELISPOT results. Our data are consistent with those of a previous report suggesting that specific T-cells control BKV latency before transplantation, and in this way may influence BKV reactivation after transplantation (40). These results suggest that low BKV-specific cellular responses before transplantation can be used as a risk factor to predict the development of BK viremia after transplantation. Regarding the target antigens of BKV-specific cellular immunity, our data did not identify the dominant target antigen. This finding confirms previous reports that it is not clear whether certain BKV peptides have a more dominant antigenicity (36, 41).

Given both BKV-IgG levels and BKV-ELISPOT results were correlated with BK viremia (> 10^4^ copies/mL of BKV-DNA detected during the follow-up period), we analyzed the diagnostic performance of a combination of these biomarkers. Among KTRs who exhibited low levels of anti-BKV-IgG and low total BKV-ELISPOT results, and who received a kidney from the D-H group, the PPV and NPV for BKV viremia after transplantation were found to be 41.7% and 88.9% respectively. The relationship between impairment of BKV-specific cellular immunity and increased BKV replication in BK viremia remains uncertain (39). In a previous study, impaired functionality of cytomegalovirus (CMV) or BKV-specific T cells was evident in some transplant recipients for a prolonged period even after virus control (42–44). In present study, we used threshold of 10^4^ copies/mL for BK viremia and initiation of therapeutic intervention. However, several studies have explored alternative thresholds for initiating therapy to prevent the progression to BKVAN. For instance, Hassan et al. reported that the currently suggested plasma viral load cutoff of 10^4^ copies/mL for BK virus tends to underestimate the diagnosis of BKVAN (45). Another study by Hirsch et al. also indicated that viral nephropathy was linked to plasma viral loads of 7.7×10^3^ copies/mL or higher (28). Randhawa et al. emphasized customized thresholds for medical centers, given their significant impact on predictive BKV infection outcomes (46). When we reexamined the dataset using a threshold of 10^3^ copies/mL for BK viremia, two additional patients were reclassified into the BK viremia group. Consequently, the association between donor BKV-IgG levels and BK viremia (> 10^3^ copies/mL) after KT was also significant (P value = 0.001). Similarly, BKV-specific ELISPOT results showed comparable diagnostic performances between data using thresholds of 10^3^ copies/mL and 10^4^ copies/mL (**Supplemental Table 1**). It is very important to establish risk factors for BKV infection after transplantation and application of proper BKV viral load cutoff, because careful monitoring of serum BKV and early reduction of immunosuppressants can resolve BK viremia and prevent transplant failure due to BKVAN in transplanted kidneys (47). Close monitoring after transplantation is necessary for patients whose donors showed high absorbance before kidney transplantation. Our results indicate anti-BKV-antibody-negative patients with a low ELISPOT results are expected to be at greater risk of BKV reactivation, so thorough monitoring is required. Thus, the application of pretransplant screening tests using anti-BKV-IgG and ELISPOT assays in conjunction with assessment of posttransplant blood BKV-DNA viral load may provide more accurate guidance for therapeutic intervention in KTRs with BKV infection.

This study had some limitations. First, a prospective cohort analysis was conducted at a single center including relatively small number of patients. Second, only five of 14 BK viremia (+) patients developed BKVAN. Therefore, our study could not determine whether pretransplant anti-BKV-IgG and BK-ELISPOT results can identify KTRs who will develop BKVAN. Finally, anti-BKV-IgG levels were not validated in this study. For clinical application, cut-off values should be investigated with standard methods to stratify individual risk of BKV infection. In addition, our study used a commercially available anti-BKV IgG kit targeting the BKV serotype I and IV genotypes. Therefore, applying our results to other BKV serotypes could potentially introduce variability. Follow-up investigations focusing on all BKV serotypes are essential to comprehensively assess clinical significance of BKV serostatus. Well-designed prospective studies controlling for relevant confounding factors are needed before using anti-BKV-IgG and BKV-ELISPOT assays clinically to predict posttransplant BKV infection. Despite these limitations, our study focused on BKV serostatus and BKV-ELISPOT results before transplantation and demonstrated the diagnostic value of combining these tests. In conclusion, pretransplant donor and recipient anti-BKV-IgG levels and BKV-ELISPOT assay results may be used to identify patients at risk of BKV infection. Further studies are needed to confirm our results and validate the anti-BKV-IgG cut-off levels and BKV-ELISPOT assays as tools to predict post-kidney-transplant BKV infection.

## Data availability statement

The raw data supporting the conclusions of this article will be made available by the authors, without undue reservation.

## Ethics statement

The studies involving humans were approved by Institutional Review Board at Seoul St. Mary’s Hospital (KC19TESI0043). The studies were conducted in accordance with the local legislation and institutional requirements. The human samples used in this study were acquired from a by- product of routine care or industry. Written informed consent for participation was not required from the participants or the participants’ legal guardians/next of kin in accordance with the national legislation and institutional requirements.

## Author contributions

HB performed the experiments and wrote the paper. HB and SJ analyzed the data. E-JO designed the study and edited the paper. BC and CY supervised specimen selection and the collection of clinical information. All authors contributed to the article and approved the submitted version.

## References

[1] Kean JM Rao S Wang M Garcea RL. Seroepidemiology of human polyomaviruses. PloS Pathog (2009) 5:e1000363. doi: 10.1371/journal.ppat.1000363 19325891 PMC2655709

[2] Wunderink HF van der Meijden E van der Blij-de Brouwer CS Mallat MJ Haasnoot GW van Zwet EW. Pretransplantation donor-recipient pair seroreactivity against BK polyomavirus predicts viremia and nephropathy after kidney transplantation. Am J Transplant (2017) 17:161–72. doi: 10.1111/ajt.13880 27251361

[3] Hirsch HH Randhawa P. BK polyomavirus in solid organ transplantation. Am J Transplant (2013) 13 Suppl 4:179–88. doi: 10.1111/ajt.12110 23465010

[4] Fishman JA. BK virus nephropathy–polyomavirus adding insult to injury. N Engl J Med (2002) 347:527–30. doi: 10.1056/NEJMe020076 12181409

[5] Hirsch HH Randhawa PS Practice ASTIDCo. BK polyomavirus in solid organ transplantation-Guidelines from the American Society of Transplantation Infectious Diseases Community of Practice. Clin Transplant (2019) 33:e13528. doi: 10.1111/ctr.13528 30859620

[6] Schachtner T Stein M Babel N Reinke P. The loss of BKV-specific immunity from pretransplantation to posttransplantation identifies kidney transplant recipients at increased risk of BKV replication. Am J Transplant (2015) 15:2159–69. doi: 10.1111/ajt.13252 25808077

[7] Udomkarnjananun S Kerr SJ Francke MI Avihingsanon Y van Besouw NM Baan CC. A systematic review and meta-analysis of enzyme-linked immunosorbent spot (ELISPOT) assay for BK polyomavirus immune response monitoring after kidney transplantation. J Clin Virol (2021) 140:104848. doi: 10.1016/j.jcv.2021.104848 33979739

[8] Mutlu E Koksoy S Mutlu D Yilmaz VT Kocak H Dinckan A. Quantitative analysis of BKV-specific CD4+ T cells before and after kidney transplantation. Transpl Immunol (2015) 33:20–6. doi: 10.1016/j.trim.2015.05.005 26048051

[9] Miettinen J Lautenschlager I Weissbach F Wernli M Auvinen E Mannonen L. BK polyomavirus viremia and antibody responses of pediatric kidney transplant recipients in Finland. Pediatr Transplant (2019) 23:e13324. doi: 10.1111/petr.13324 30447046

[10] Dakroub F Touzé A Sater FA Fiore T Morel V Tinez C. Impact of pre-graft serology on risk of BKPyV infection post-renal transplantation. Nephrol Dialysis Transplant (2021) 37:781–8. doi: 10.1093/ndt/gfab279 34586413

[11] Bae H Na DH Chang JY Park KH Min JW Ko EJ. Usefulness of BK virus-specific interferon-γ enzyme-linked immunospot assay for predicting the outcome of BK virus infection in kidney transplant recipients. Korean J Intern Med (2021) 36:164–74. doi: 10.3904/kjim.2019.339 PMC7820663 32241081

[12] Solis M Velay A Porcher R Domingo-Calap P Soulier E Joly M. Neutralizing antibody-mediated response and risk of BK virus-associated nephropathy. J Am Soc Nephrol (2018) 29:326–34. doi: 10.1681/asn.2017050532 PMC5748919 29042457

[13] Dakroub F Touzé A Akl H Brochot E. Pre-transplantation assessment of BK virus serostatus: significance, current methods, and obstacles. Viruses (2019) 11:945. doi: 10.3390/v11100945 31615131 PMC6833059

[14] Sood P Senanayake S Sujeet K Medipalli R Van-Why SK Cronin DC. Donor and recipient BKV-specific IgG antibody and posttransplantation BKV infection: a prospective single-center study. Transplantation (2013) 95:896–902. doi: 10.1097/TP.0b013e318282ba83 23511214

[15] Schmitt C Raggub L Linnenweber-Held S Adams O Schwarz A Heim A. Donor origin of BKV replication after kidney transplantation. J Clin Virol (2014) 59:120–5. doi: 10.1016/j.jcv.2013.11.009 24361208

[16] Saláková M Ludvíková V Hamšíková E Kolářová M Šroller V Viklický O. Pretransplantation seroreactivity in kidney donors and recipients as a predictive factor for posttransplant BKPyV-DNAemia. Front Immunol (2022) 13:929946. doi: 10.3389/fimmu.2022.929946 35967393 PMC9364833

[17] Ginevri F De Santis R Comoli P Pastorino N Rossi C Botti G. Polyomavirus BK infection in pediatric kidney-allograft recipients: a single-center analysis of incidence, risk factors, and novel therapeutic approaches. Transplantation (2003) 75:1266–70. doi: 10.1097/01.Tp.0000061767.32870.72 12717214

[18] Smith JM McDonald RA Finn LS Healey PJ Davis CL Limaye AP. Polyomavirus nephropathy in pediatric kidney transplant recipients. Am J Transplant (2004) 4:2109–17. doi: 10.1111/j.1600-6143.2004.00629.x 15575916

[19] Zhou W Sharma M Martinez J Srivastava T Diamond DJ Knowles W. Functional characterization of BK virus-specific CD4+ T cells with cytotoxic potential in seropositive adults. Viral Immunol (2007) 20:379–88. doi: 10.1089/vim.2007.0030 17931108

[20] Zareei N Miri HR Karimi MH Afshari A Geramizadeh B Roozbeh J. Increasing of the interferon-gamma gene expression during polyomavirus BK infection in kidney transplant patients. Microb Pathog (2019) 129:187–94. doi: 10.1016/j.micpath.2019.02.015 30769026

[21] Schaenman JM Korin Y Sidwell T Kandarian F Harre N Gjertson D. Increased frequency of BK virus-specific polyfunctional CD8+ T cells predict successful control of BK viremia after kidney transplantation. Transplantation (2017) 101:1479–87. doi: 10.1097/tp.0000000000001314 PMC5219876 27391197

[22] Comoli P Basso S Azzi A Moretta A De Santis R Del Galdo F. Dendritic cells pulsed with polyomavirus BK antigen induce ex vivo polyoma BK virus-specific cytotoxic T-cell lines in seropositive healthy individuals and renal transplant recipients. J Am Soc Nephrol (2003) 14:3197–204. doi: 10.1097/01.asn.0000096374.08473.e3 14638918

[23] Dekeyser M De Goër M-G Hendel Chavez H Boutin E Herr F Lhotte R. Heterospecific immunity help to sustain an effective BK-virus immune response and prevent BK-virus-associated nephropathy. researchSquare [Preprint] (2022). doi: 10.21203/rs.3.rs-1650939/v1

[24] Balba GP Javaid B Timpone JG Jr. BK polyomavirus infection in the renal transplant recipient. Infect Dis Clin North Am (2013) 27:271–83. doi: 10.1016/j.idc.2013.02.002 23714340

[25] Kuypers DR. Management of polyomavirus-associated nephropathy in renal transplant recipients. Nat Rev Nephrol (2012) 8:390–402. doi: 10.1038/nrneph.2012.64 22508181

[26] Kwak EJ Vilchez RA Randhawa P Shapiro R Butel JS Kusne S. Pathogenesis and management of polyomavirus infection in transplant recipients. Clin Infect Dis (2002) 35:1081–7. doi: 10.1086/344060 12384842

[27] Kahan AV Coleman DV Koss LG. Activation of human polyomavirus infection — Detection by cytologic technics. Am J Clin Pathol (1980) 74:326–32. doi: 10.1093/ajcp/74.3.326 6251715

[28] Hirsch HH Knowles W Dickenmann M Passweg J Klimkait T Mihatsch MJ. Prospective study of polyomavirus type BK replication and nephropathy in renal-transplant recipients. N Engl J Med (2002) 347:488–96. doi: 10.1056/NEJMoa020439 12181403

[29] Andrews CA Daniel RW Shah KV. Serologic studies of papovavirus infections in pregnant women and renal transplant recipients. Prog Clin Biol Res (1983) 105:133–41. 6304749

[30] Bohl DL Storch GA Ryschkewitsch C Gaudreault-Keener M Schnitzler MA Major EO. Donor origin of BK virus in renal transplantation and role of HLA C7 in susceptibility to sustained BK viremia. Am J Transplant (2005) 5:2213–21. doi: 10.1111/j.1600-6143.2005.01000.x 16095500

[31] Dadhania D Snopkowski C Ding R Muthukumar T Chang C Aull M. Epidemiology of BK virus in renal allograft recipients: independent risk factors for BK virus replication. Transplantation (2008) 86:521–8. doi: 10.1097/TP.0b013e31817c6447 PMC3647687 18724220

[32] Bohl DL Brennan DC Ryschkewitsch C Gaudreault-Keener M Major EO Storch GA. BK virus antibody titers and intensity of infections after renal transplantation. J Clin Virol (2008) 43:184–9. doi: 10.1016/j.jcv.2008.06.009 PMC2701220 18676176

[33] Prosser SE Orentas RJ Jurgens L Cohen EP Hariharan S. Recovery of BK virus large T-antigen-specific cellular immune response correlates with resolution of bk virus nephritis. Transplantation (2008) 85:185–92. doi: 10.1097/TP.0b013e31815fef56 18212622

[34] Schachtner T Muller K Stein M Diezemann C Sefrin A Babel N. BK virus-specific immunity kinetics: a predictor of recovery from polyomavirus BK-associated nephropathy. Am J Transplant (2011) 11:2443–52. doi: 10.1111/j.1600-6143.2011.03693.x 21831150

[35] Trydzenskaya H Sattler A Müller K Schachtner T Dang-Heine C Friedrich P. Novel approach for improved assessment of phenotypic and functional characteristics of BKV-specific T-cell immunity. Transplantation (2011) 92:1269–77. doi: 10.1097/TP.0b013e318234e0e5 22124284

[36] Binggeli S Egli A Schaub S Binet I Mayr M Steiger J. Polyomavirus BK-specific cellular immune response to VP1 and large T-antigen in kidney transplant recipients. Am J Transplant (2007) 7:1131–9. doi: 10.1111/j.1600-6143.2007.01754.x 17359507

[37] Leboeuf C Wilk S Achermann R Binet I Golshayan D Hadaya K. BK polyomavirus-specific 9mer CD8 T cell responses correlate with clearance of BK viremia in kidney transplant recipients: first report from the Swiss transplant cohort study. Am J Transplant (2017) 17:2591–600. doi: 10.1111/ajt.14282 28326672

[38] Schachtner T Stein M Sefrin A Babel N Reinke P. Inflammatory activation and recovering BKV-specific immunity correlate with self-limited BKV replication after renal transplantation. Transpl Int (2014) 27:290–301. doi: 10.1111/tri.12251 24279642

[39] Schmidt T Adam C Hirsch HH Janssen MW Wolf M Dirks J. BK polyomavirus-specific cellular immune responses are age-dependent and strongly correlate with phases of virus replication. Am J Transplant (2014) 14:1334–45. doi: 10.1111/ajt.12689 24726000

[40] Šťastná-Marková M Hamšíková E Hainz P Hubáček P Kroutilová M Kryštofová J. Pretransplant BK Virus-Specific T-Cell-Mediated Immunity and Serotype Specific Antibodies May Have Utility in Identifying Patients at Risk of BK Virus-Associated Haemorrhagic Cystitis after Allogeneic HSCT. Vaccines (Basel) (2021) 9(11):1226. doi: 10.3390/vaccines9111226 34835157 PMC8625163

[41] Mueller K Schachtner T Sattler A Meier S Friedrich P Trydzenskaya H. BK-VP3 as a new target of cellular immunity in BK virus infection. Transplantation (2011) 91:100–7. doi: 10.1097/tp.0b013e3181fe1335 21452414

[42] Papadopoulou A Koukoulias K Alvanou M Papadopoulos VK Bousiou Z Kalaitzidou V. Patient risk stratification and tailored clinical management of post-transplant CMV-, EBV-, and BKV-infections by monitoring virus-specific T-cell immunity. EJHaem (2021) 2:428–39. doi: 10.1002/jha2.175 PMC9175754 35844677

[43] Lee H Oh E-J. Laboratory diagnostic testing for cytomegalovirus infection in solid organ transplant patients. Korean J Transplant (2022) 36:15–28. doi: 10.4285/kjt.22.0001 35769434 PMC9235525

[44] Lee H Park KH Ryu JH Choi AR Yu JH Lim J. Cytomegalovirus (CMV) immune monitoring with ELISPOT and QuantiFERON-CMV assay in seropositive kidney transplant recipients. PloS One (2017) 12:e0189488. doi: 10.1371/journal.pone.0189488 29232714 PMC5726762

[45] Hassan S Mittal C Amer S Khalid F Patel A Delbusto R. Currently recommended BK virus (BKV) plasma viral load cutoff of ≥4 log10/mL underestimates the diagnosis of BKV-associated nephropathy: a single transplant center experience. Transpl Infect Dis (2014) 16:55–60. doi: 10.1111/tid.12164 24283677

[46] Randhawa P Ho A Shapiro R Vats A Swalsky P Finkelstein S. Correlates of quantitative measurement of BK polyomavirus (BKV) DNA with clinical course of BKV infection in renal transplant patients. J Clin Microbiol (2004) 42:1176–80. doi: 10.1128/jcm.42.3.1176-1180.2004 PMC356850 15004071

[47] Ali AM Gibson IW Birk P Blydt-Hansen TD. Pretransplant serologic testing to identify the risk of polyoma BK viremia in pediatric kidney transplant recipients. Pediatr Transplant (2011) 15:827–34. doi: 10.1111/j.1399-3046.2011.01583.x 22111998

