# Supplementary figures and images for: Pretransplant BKV-IgG serostatus and BKV-specific ELISPOT assays to predict BKV infection after kidney transplantation
Source: Front Immunol. 2023 Sep 21;14:1243912. doi: 10.3389/fimmu.2023.1243912 (PMC10551174; doi:10.3389/fimmu.2023.1243912)

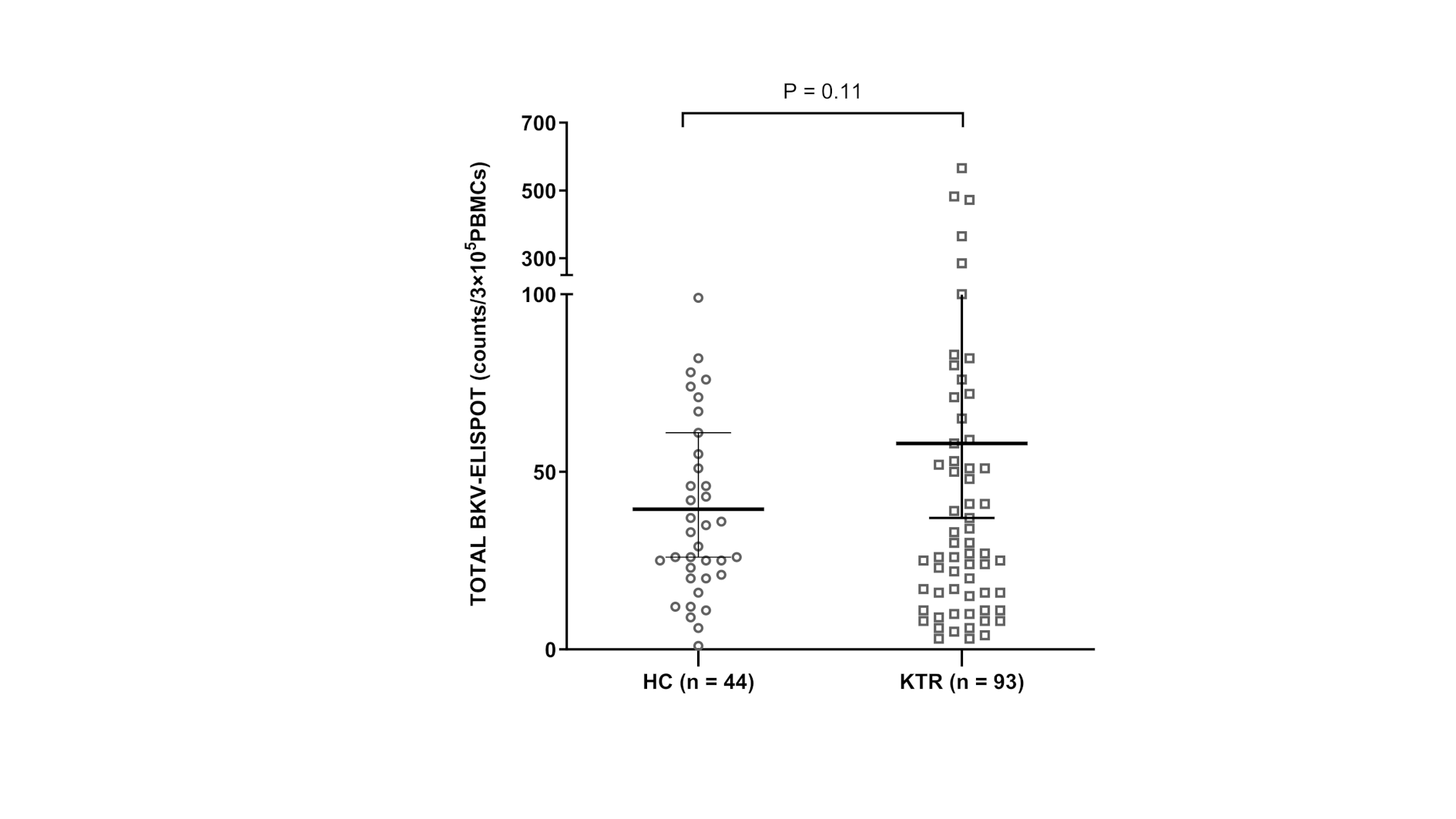

Supplement: Supplementary file 1 [file Image_1.tif]

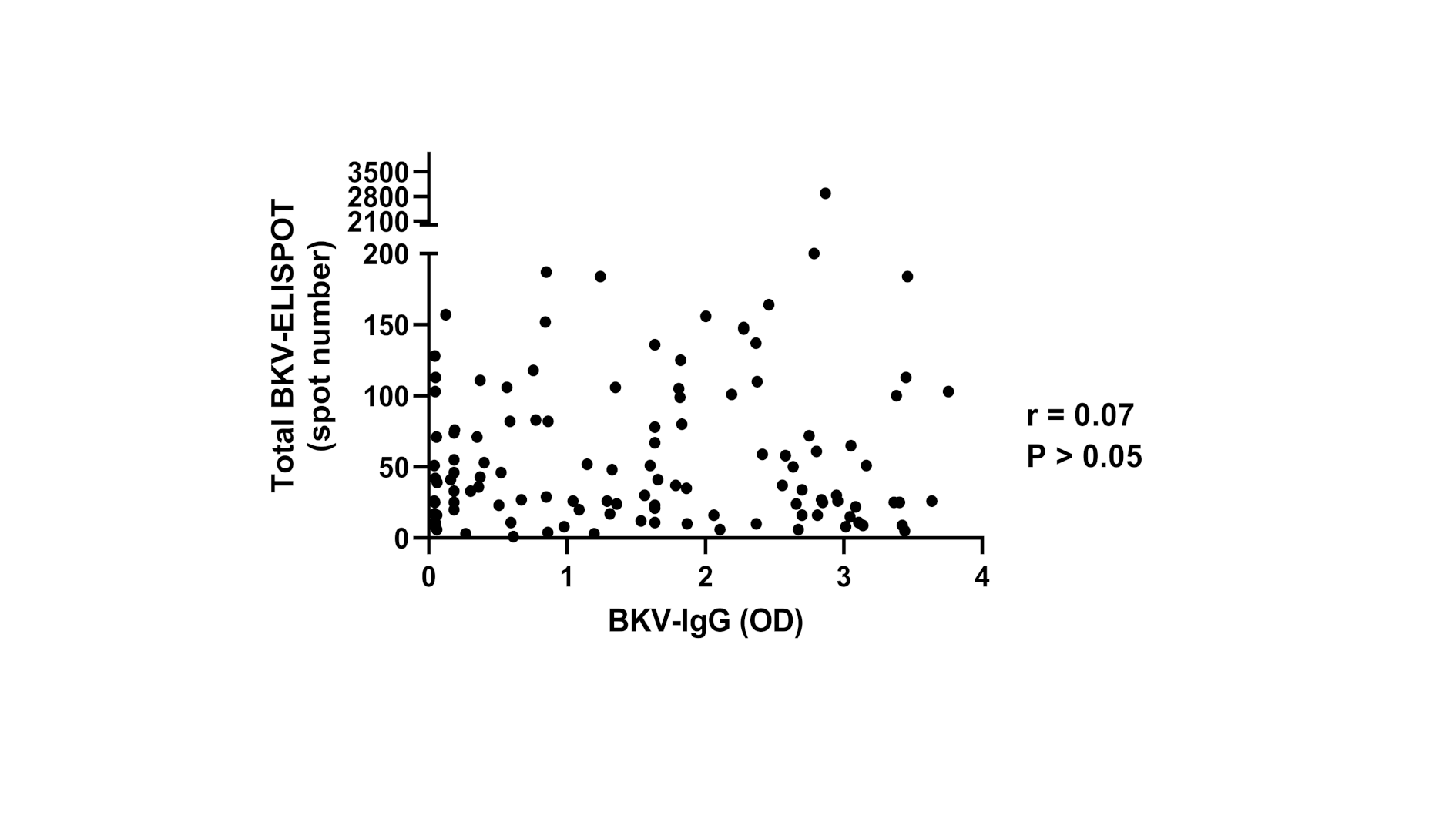

Supplement: Supplementary file 2 [file Image_2.tif]
