# Supplementary material for: Pretransplant BKV-IgG serostatus and BKV-specific ELISPOT assays to predict BKV infection after kidney transplantation
Source: Front Immunol. 2023 Sep 21;14:1243912. doi: 10.3389/fimmu.2023.1243912 (PMC10551174; doi:10.3389/fimmu.2023.1243912)
Supplement: Supplementary file 3 [file Table_1.docx]

**Supplemental Table 1.** Diagnostic accuracy of pre-transplant donor and recipient anti-BKV-IgG levels and recipient BKV-ELISPOT results in kidney transplant recipients for prediction of BK viremia (>10^3^ copies/mL) after kidney transplantation in the study population.

|  | Sensitivity  % (95% CI) | Specificity  % (95% CI) | PPV  % (95% CI) | NPV  % (95% CI) |
| --- | --- | --- | --- | --- |
| High donor anti-BKV-IgG | 87.5  (60.4-97.8) | 57.1  (45.4-68.2) | 29.8  (23.6-36.8) | 95.7  (85.6-98.8) |
| Low recipient BKV-ELISPOT* | 68.8  (41.3-89.0) | 54.6  (42.8-65.9) | 23.9  (17.2-32.2) | 89.4  (79.8-94.7) |
| High donor anti-BKV-IgG and low recipient BKV-ELISPOT | 56.3  (29.9-80.2) | 80.5  (69.6-88.7) | 37.5  (24.3-52.9) | 89.9  (83.4-94.0) |
| High donor anti-BKV-IgG, and low recipient anti-BKV-IgG and BKV-ELISPOT | 37.5  (15.2-64.6) | 92.2  (83.8-97.1) | 50.0  (27.0-73.0) | 87.7  (82.9-91.3) |
| High donor anti-BKV-IgG or low recipient BKV-ELISPOT | 100.0  (79.4-100) | 31.2  (21.1-42.7) | 23.2  (20.6-26.0) | 100.0  (85.8-100.0) |

*total BKV-ELISPOT result with spot number ≤ 53 / 3 x 105 PBMCs

Abbreviations: PPV, positive predictive value; NPV, negative predictive value; CI, confidence interval
